# Increased prefrontal cortex interleukin-2 protein levels and shift in the peripheral T cell population in progressive supranuclear palsy patients

**DOI:** 10.1038/s41598-019-44234-y

**Published:** 2019-05-23

**Authors:** Rasmus Rydbirk, Betina Elfving, Jonas Folke, Bente Pakkenberg, Kristian Winge, Tomasz Brudek, Susana Aznar

**Affiliations:** 1 Research Laboratory for Stereology and Neuroscience, Bispebjerg-Frederiksberg Hospital, University Hospital of Copenhagen, Nielsine Nielsens Vej 6B, stair 11B, 2nd floor, DK-2400 Copenhagen NW, Denmark; 2 Translational Neuropsychiatry Unit, Department of Clinical Medicine, Aarhus University, Skovagervej 2, DK-8240 Risskov, Denmark; 3 Institute of Clinical Medicine, Faculty of Health, University of Copenhagen, Blegdamsvej 3B, DK-2200 Copenhagen, Denmark; 4 Department of Neurology, Bispebjerg-Frederiksberg Hospital, University Hospital of Copenhagen, Ebba Lunds Vej 44, DK-2400 Copenhagen NW, Denmark

**Keywords:** Neurodegenerative diseases, Neurodegenerative diseases

## Abstract

Accumulating evidence suggests neuroinflammation to be an integrated feature of neurodegeneration. Profiling inflammatory mediators across diseases may reveal common and disease-specific signatures. Here, we focused on progressive supranuclear palsy (PSP), a tauopathy presenting motor and cognitive dysfunction. We screened for 21 cytokines and growth factors in the dorsomedial prefrontal cortex of 16 PSP and 16 control brains using different quantitative techniques. We found and validated increased interleukin (IL)-2 protein levels in the PSP group expressed locally by neurons and glia cells. We further investigated central players in neuroinflammatory pathways and found increased mRNA expression of glycogen synthase kinase 3 beta (*GSK3B*). IL-2 and GSK3B proteins are T and natural killer (NK) cell regulators and have previously been associated with other neurodegenerative diseases such as Alzheimer’s disease, Parkinson’s disease and multiple system atrophy. In addition, we identified a shift in peripheral CD4^+^ and CD8^+^ T cell populations toward increased numbers of memory and reduced numbers of naive T cells. We also observed increased numbers of CD56^+^ NK cells, but not of CD56^+^CD57^+^ or CD57^+^ NK cells. Our findings suggest a role for IL-2 in PSP disease processes and point toward active and possibly dysfunctional peripheral immune responses in these patients.

## Introduction

Progressive supranuclear palsy (PSP) is a progressive, neurodegenerative disease that shares clinical features with other parkinsonian disorders and Alzheimer’s disease (AD)^1^. Neuropathologically, PSP is characterized by accumulation of tau protein in neurofibrillary tangles (NFT) in tufted astrocytes. The highest load of NFTs are localized in different areas of the basal ganglia and the brainstem, but cortical areas including the frontal lobe are also affected^2,3^. Brain imaging studies have shown that brain atrophy in PSP patients is accompanied by microglial activation in the frontal cortex^4,5^. This suggests that neuroinflammatory processes are part of the course of the disease. Still, there is a knowledge gap regarding how neuroimmunomodulating factors are affected in PSP brains. The few existing studies approaching this topic have focused solely on gene expression without reporting protein levels^6,7^. This information is important, not only for understanding the pathophysiology behind this disease, but also for illustrating to what extend neuroinflammatory processes are a common manifestation in neurodegenerative diseases. To our knowledge, no study has investigated the immune cell profile in PSP patients to shed light on the possible involvement of the peripheral immune system in PSP.

The primary aim of the present study was to investigate the neuroinflammatory signaling profile in the dorsomedial prefrontal cortex (dmPFC) of PSP patients. Using multiplex assays, we screened for protein levels of 21 cytokines and growth factors in post-mortem brain samples from 16 PSP patients and 16 normal controls (NCs). Significant differences were validated using sensitive electrochemiluminescence technology. To depict the underlying processes associated with cytokine aberrancies, we performed quantitative gene expression analyses of up- and down-stream targets as well as of signaling effector molecules. Lastly, using flow cytometry we screened for peripheral changes in CD4^+^ and CD8^+^ T cell, and NK cell numbers in PSP patients.

## Results

### IL-2 protein levels are increased in PSP patients

**Figure 1:**
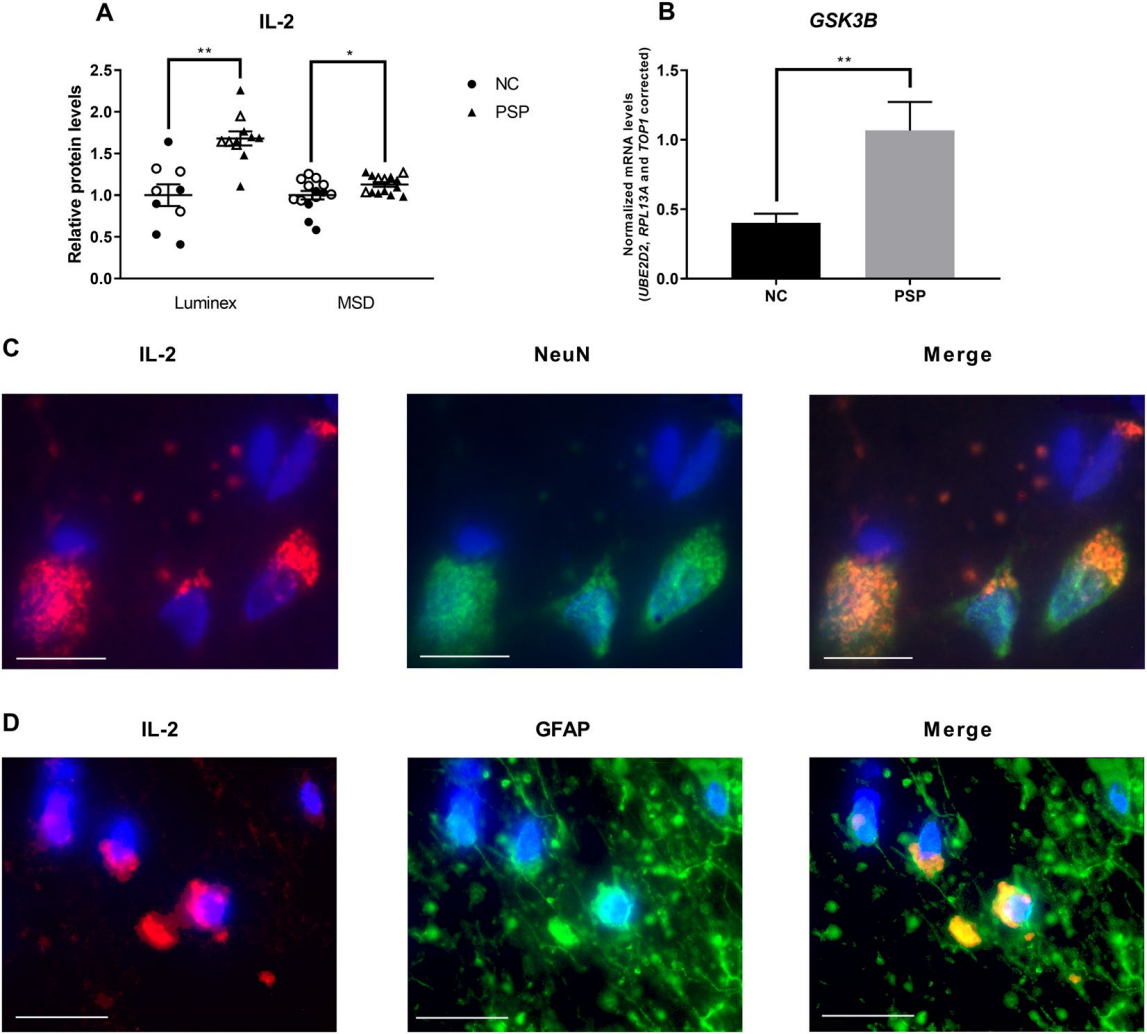
Interleukin-2 (IL-2) and *glycogen synthase kinase 3 beta* (*GSK3B*) levels. (**A**) IL-2 protein levels in progressive supranuclear palsy (PSP) patients normalized to normal control (NC) levels. Protein levels were measured using two commercial assays based on Luminex or MSD technology. Filled symbols mark male subjects, open symbols mark female subjects. (**B**) mRNA levels of *GSK3B*. Expression levels normalized to the reference genes ubiquitin-conjugating enzyme 2D2 (*UBE2D2*), ribosomal protein 13a (*RPL13A*) and DNA topoisomerase 1 (*TOP1*). Data presented as mean ± SEM. *p < 0.050; **p < 0.010. (**C**) Immunofluorescent stainings of IL-2 (red), and NeuN (green) or (**D**) GFAP (green). Nuclei are stained with DAPI (blue). Scale bars: 20 µm, 100X magnification.

Of the 21 protein targets, IL-2 (F(4,15) = 7.931, *p* = 0.001, *R*^2^ = 0.68) and G-CSF (F(4,11) = 16.141, *p* < 0.001, *R*^2^ = 0.85) passed the correction for multiple testing, and both cytokines were predicted by group (t = 3.888, *p* = 0.002 and t = −7.819, *p* < 0.001, respectively). We then validated these findings using a more sensitive singleplex assay. The observed differences in G-CSF protein levels did not pass validation (F(4,11) = 0.501, *p* > 0.050, *R*^2^ = 0.25) but we confirmed differences in IL-2 protein levels (F(4,23) = 3.698, *p* = 0.018, *R*^2^ = 0.39; Fig. 1A). PSP patients showed increased IL-2 protein levels (t = 3.425, *p* = 0.002), while in general, males showed lower levels than females (t = −2.804, *p* = 0.010). Scores for the presence of dementia and resting tremor (Table 1) within the first year of diagnosis correlated with Luminex measurements of IL-2 protein levels (Spearman’s rho(ρ) = −0.881, *p* < 0.001, and ρ = 0.798, *p* = 0.006, respectively; Supplementary Fig. S3), with dementia scores showing a negative correlation whereas tremor scores showed a positive correlation. Conversely, no correlations were found with MSD measurements of IL-2 protein levels (*p* > 0.050; Supplementary Fig. S3). No other clinical scores correlated significantly to IL-2 protein levels (*p* > 0.050; data not shown).

**Table 1:** Clinical characteristics of the PSP patients.

Patient ID	1	2	3	4	5	6	7	8	9	10	11	12	13
Disease duration (years)	5	8	5.5	8	10	5	9	9	6	10	12	7	12
Gaze palzy at onset	Yes	Yes	Yes	Yes	No	Yes	No	Yes	Yes	No	No	Yes	Yes
Dementia*	No	Yes	Yes	Yes	Yes	Yes	No	Yes	Yes	Yes	Yes	Yes	Yes
Cognitive dysfunction*^a^	+	+++	+	++	+++	++	+	+	+++	++	+++	+	+++
Apathy*^a^	+	−	−	+	+++	−	−	−	+++	−	+	+	−
Speech impairment*^a^	+	+++	++	+	++	+	++	+++	++	+	+	+++	+++
Sleep problems*^a^	++	++	+	++	+	++	+++	+++	+++	−	++	++	++
Nocturia*^a^	++	++	++	++	++	++	++	++	+++	−	+++	+++	+
Response to L-DOPA*^b^	+	−	+	−	+	−	++	+	−	−	++	+	+
Resting tremor*	Yes	No	No	No	No	No	Yes	No	No	Yes	No	No	No
Axial rigidity*^a^	+	++	++	+	+	+	+++	+	+	+	+	+	++
Bradykinesia*^a^	+	+	++	++	+++	+++	+	+	+++	+	++	+++	+++
Postural instability at onset	Yes	Yes	Yes	Yes	Yes	Yes	Yes	Yes	No	No	Yes	Yes	No
Diagnosis	PSP-P	PSP-RS	PSP-RS	PSP-RS	PSP-F	PSP-RS	PSP-P	PSP-SL	PSP-F	PSP-RS	PSP-P/RS	PSP-P/RS	PSP-F

Data are shown for the samples donated by Bispebjerg Brain Bank, from which medical records were available. *Based on the first year after diagnosis; ^a^+: Diminishable, ++: Some, +++: Intense; ^b^+: Poor, ++: Good; PSP-P: PSP with parkinsonism resembling Parkinson’s disease; PSP-RS: PSP with Richardson’s syndrome; PSP-F: PSP with frontal lobe cognitive or behavioural presentations; PSP-SL: PSP with speech or language disorders^1^.

### *GSK3B* mRNA levels are increased in PSP patients

We then measured mRNA levels of central protein players in inflammatory pathways (Table 2). We identified increased mRNA levels of *GSK3B* in PSP patients (F(5,17) = 4.845, *p* = 0.006, *R*^2^ = 0.59; Fig. 1B) which was predicted by group (t = 3.914, *p* = 0.001) and RIN (t = −2.200, *p* = 0.042). Further, investigating downstream targets to *GSK3B*, only the PD-related *NR4A*2 was significantly described by our model (F(5,13) = 4.413, *p* = 0.014), but only by RIN (t = −2.973, *p* = 0.011) and not group (*p* > 0.050). Neither *NFKBIA* nor *RELA*, both immune regulatory factors, were significantly described by our model (*p* > 0.050).

**Table 2:** Results from protein and mRNA measurements.

Protein	NC		PSP		Model statistics	
	Mean	SD	Mean	SD	*p*	*R* ^2^
bFGF	1132.843	337.960	960.472	387.762	0.470#	0.14
G-CSF	58.583	15.971	20.261	6.811	**<0**.**001#**	0.85
G-CSF (M)	22.810	21.688	11.726	7.201	0.501	0.24
GM-CSF	43.147	9.401	46.913	5.573	0.344#	0.20
IFN-γ	13.254	6.149	14.040	6.301	0.583#	0.23
IL-1β	1.906	0.999	1.692	1.243	**0**.038#*	0.43
IL-2	3.377	1.320	5.675	0.951	**0**.**001#**	0.68
IL-2 (M)	1.479	0.283	1.670	0.157	**0**.**018**	0.39
IL-6	16.126	13.332	8.842	5.991	0.191#	0.34
IL-7	6.768	1.607	5.588	1.585	**0**.023#*	0.42
IL-13	1.232	0.330	0.893	0.159	**0**.015#*	0.44
MCP1	15.553	7.030	14.933	6.330	0.393#	0.19
MIP1β	2.107	0.617	1.727	0.400	0.360#	0.20
PDGF-BB	32.421	11.170	34.885	14.453	0.640#	0.10
**mRNA**	**Mean**	**SD**	**Mean**	**SD**	***p***	***R*** ^***2***^
*GSK3B*	0.403	0.228	1.066	0.682	**0**.**006**	0.59
*NFKBIA*	1.905	1.957	1.049	0.570	0.553	0.20
*NR4A2*	1.922	0.846	3.257	2.985	**0**.**014**	0.63
*RELA*	1.189	0.885	0.980	0.559	0.257	0.30

All measurements are in pg/ml. bFGF: basic fibroblast growth factor; G-CSF: granulocyte colony-stimulating factor; GM-CSF: granulocyte macrophage colony-stimulating factor; IFN-γ: interferon-kappa; IL-1β, -2, -6, -7, and -13: interleukin-1beta, -2, -6, -7, and -13, respectively; MCP1: monocyte chemoattractant protein 1; MIP1β: macrophage inflammatory protein 1beta; PDGF-BB: platelet-derived growth factor-BB; *GSK3B*: glycogen synthase kinase 3 beta; *NFKBIA*: nuclear factor kappa(κ)-light-chain-enhancer of activated B cells inhibitor alpha; *NR4A2*: nuclear receptor subfamily 4 group A member 2 (also known as *Nurr1*); *RELA*: NF-κB subunit p65; *RET*: receptor tyrosine kinase rearranged during transfection. *did not pass Bonferroni correction; (M) measured on MSD instrument; # subject to multiple comparison adjustment; *R*^*2*^ alignment with model.

### IL-2 protein is expressed by NeuN^+^ and GFAP^+^ brain cells

To investigate if IL-2 is produced locally by brain cells, we performed double immunofluorescence labelling using specific antibodies against IL-2, neuronal nuclei (NeuN), and glial fibrillary acidic protein (GFAP) on brain sections from both PSP patients and NCs. We observed co-localization of IL-2 with both NeuN and GFAP in both groups (Fig. 1C,D) confirming that IL-2 is produced locally in the brain.

### The number of peripheral T and NK cells are altered in blood of PSP patients

**Figure 2:**
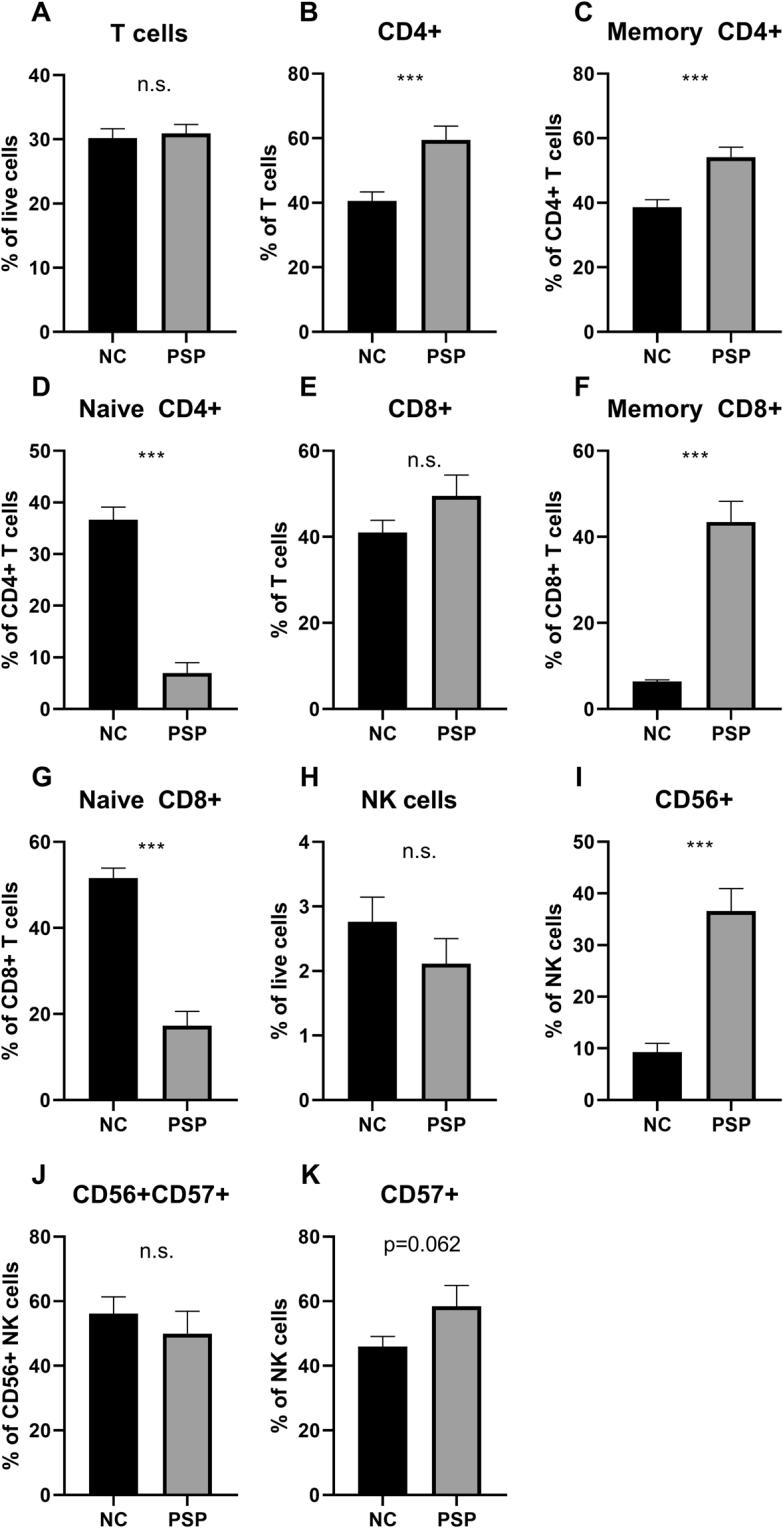
T and NK cell populations in blood of PSP patients. We investigated peripheral blood mononuclear cells in a new cohort consisting of 18 normal controls and nine PSP patients. We investigated fractions of (**A**–**G**) T cells and (**H**–**K**) natural killer (NK) cells. For gating strategies, see Supplementary Figs. 1 and 2. Data are presented as mean ± SEM; n.s. non-significant; ***p < 0.001.

Lastly, using flow cytometry on a new cohort (18 NCs, nine PSP patients) we investigated whether the elevated levels of IL-2 in PSP brains could be reflected in the composition of peripheral T lymphocytes. We observed a significant increase in numbers of CD4^+^ T cells in PSP patients (t = 3.812, *p* < 0.001; Fig. 2B) accompanied by a shift in the ratio between memory and naive CD4^+^ T cells (t = 3.915, *p* < 0.001, and t = 7.940, *p* < 0.001, respectively; Fig. 2C,D). CD8^+^ T cells numbers did not differ between the groups (t = 1.602, *p* = 0.122; Fig. 2E), however, we also observed a shift in the ratio between memory and naive CD8^+^ T cells (t = 7.667, *p* < 0.001, and t = 8.595, *p* < 0.001, respectively; Fig. 2F,G) in PSP patients. We did not see any differences in the levels of total T cells (t = 0.318, *p* = 0.754; Fig. 2A). Since IL-2 protein not only affects classical T cells but also interacts with different types of natural killer (NK) cells, we included NK cell markers in our setup. We saw no differences in the numbers of total NK cells (t = 1.071, *p* = 0.295; Fig. 2H) between the groups, however, CD56^+^ NK cell numbers were increased in PSP patients (t = 7.007, *p* < 0.001; Fig. 2I). We found a tendency towards increased CD57^+^ NK cells (t = 1.956, *p* = 0.062; Fig. 2K) whereas there was no difference in CD56^+^CD57^+^ NK cells (t = 0.699, *p* = 0.491; Fig. 2J).

## Discussion

To our knowledge, this is the first study extensively evaluating cytokine protein levels in brains affected by PSP. We screened for 18 cytokines and three growth factors and identified increased IL-2 protein levels in the prefrontal cortex of the brain in PSP patients. Generally, the effects of IL-2 are maturation and survival of T cells of the peripheral immune system^8^. However, the expression of IL-2 and IL-2 receptors is found in brain cells, and we have previously shown how NeuN^+^ cells express IL-2 protein in both healthy brains, and brains from patients diagnosed with Parkinson’s disease or multiple system atrophy^9^. Concordantly, in this study we observed co-localization of IL-2 inside both NeuN^+^ and GFAP^+^ cells. IL-2 protein has been suggested to be a neurokine^10^. Hence, the increase in IL-2 protein levels could be of neuronal origin and caused by disease processes. In the current study, for IL-2 protein levels, we observed both sex-specific differences and correlations to clinical scores. To our knowledge, no study has identified sex-specific differences in PSP, nor does the incidence rate differ between sexes^11^. The correlations to the clinical scores were significant for the Luminex measurements, however, this was not replicated for the MSD measurements. This is probably due to the nature of both methods and needs further validation. These observations might be relevant for evaluation of the clinical aspects of changes in brain IL-2 levels. Nevertheless, the current study was not designed to address such issues as the sample size is a limiting factor. Due to our modest sample size included in this experimental setup, additional studies using larger cohorts are needed to validate these findings.

One regulator of IL-2 and T cell proliferation is GSK3B protein^12^. We identified increased mRNA levels of *GSK3B* in PSP brains. In the brain, GSK3B protein participates in the production of pro-inflammatory mediators secreted by microglia cells^13^. GSK3B has previously received considerable attention in relation to PSP as GSK3B protein activity mediates aggregation of tau^14^. However, interventional strategies targeting GSK3B have been unsuccessful^15^. Although mRNA and protein levels are not directly correlated, our findings are in addition to the previous observations of a central role of GSK3B in the brains of PSP patients.

For the first time, we report on lymphocyte populations in blood of PSP patients. IL-2 protein is produced by CD4^+^ and CD8^+^ T cells as well as CD56^+^ and CD57^+^ NK cells either as developing or mature cells^8,16,17^. Overall, the observed shift in the pool of T cells toward an increase in CD45RA^−^CD45RO^+^ memory T cells may be indicative of an active, adaptive immune response. Further, the concurrent decrease in CD45RA^+^CD45RO^−^ naive T cells, accompanied by increased numbers of CD4^+^, but not CD8^+^, T cells, could be indicative of a possible reduced capacity of the adaptive immune system toward novel stimuli^18^. Lastly, the observed increase in CD56^+^ NK cells, but not of activated CD56^+^CD57^+^ NK cells, points toward an increased capacity to respond to innate immune challenges. These results are supported by the results from Santiago and Potashkin^19^ reporting on affected gene clusters in PSP that are overrepresented in biological pathways of both leukocyte and lymphocyte activation. Nevertheless, limitations to our study are the low number of individuals as well as the lack of demographic and clinical information for factors affecting peripheral lymphocyte levels. Therefore, we encourage that our observations should be validated in a separate study using a larger and well-defined cohort.

Lately, IL-2 has received much attention as an immune effector in AD since animal studies have shown a beneficial effect of IL-2 on amyloid pathology^20,21^. We could therefore speculate that the increased levels observed in the PSP brains are reflecting compensatory effects to the disease progression rather than necessarily being the cause of the detrimental effects. Therefore, IL-2 treatment aiming at specifically expanding and activating regulatory T cells recently proposed in AD^22,23^ could also be an interesting venue to explore in relation to PSP, which at present is a disease without curative treatment.

Other studies have reported on cytokine gene expression in several brain areas of PSP patients with differing results. One study observed increased expression of IL-1beta(β) in the substantia nigra and no change in the expression of transforming growth factor β in the frontal cortex of PSP patients^7^, whereas another study identified increased expression of the latter in the frontal cortex of PSP patients compared to controls^6^. In the current study, we did not assess protein levels of transforming growth factor β, nor did we investigate cytokine expression in the substantia nigra. Here, we focused on the prefrontal cortex which is an area that is only mildly affected in PSP^4^. Our aim was to identify disease pathology distant to the epicenter of the disease that may reflect earlier stages of degenerative processes. This is a limiting factor to our study, and we can therefore not exclude that cytokine expression may be different in other more affected brain areas in PSP patients.

To conclude, in the present study we have identified and validated an increase in IL-2 protein levels in the prefrontal cortex of PSP patients compared with NCs. PSP thereby presents a similar cytokine profile as the one identified for both Parkinson’s disease and multiple system atrophy in the same brain area. Further, we have observed changes in the peripheral immune system. Our results are indicative of an active and dysregulated adaptive and innate immune response. These data warrant future investigations into the possible role of the immune systems in PSP pathophysiology.

## Materials and Methods

### Patient material

The tissue for the present study originated from donated human brains. All donors provided written informed consent prior to death. PSP samples were analyzed alongside NC samples^9^. Brain samples were provided by the Bispebjerg Brain Bank (University Hospital of Copenhagen, Bispebjerg Hospital, DNK), the Netherlands Brain Bank (Netherlands Institute for Neuroscience, NLD), and the Harvard Brain Tissue Resource Center (Harvard Medical School Teaching Hospital, USA). A summary of demographic data for the 16 NC and 16 PSP brain samples are shown in Table 3. All brains were neuropathologically examined to verify the clinical diagnosis^1,24^. Medical records were available for 13 of the 16 patients. The clinical characteristics are shown in Table 1. Clinical scorings were based on the clinicians’ notes as well as appropriate, clinical tests, e.g., the Mini-Mental State Examination, the Montreal Cognitive Assessment, or the Addenbrooke's Cognitive Examination tests for dementia and cognitive dysfunction. All brain samples have been collected and handled in accordance with Danish ethical standards of the Brain Bank and the Danish Health and Medicine Authorities. Samples were stored at −80 °C prior to handling. Flow cytometry was performed on a new cohort of samples, that included 18 NC and nine PSP samples, a summary of the demographic data are shown in Table 3. These samples were generously donated by Bispebjerg Movement Disorder Biobank. This project was approved by the Regional Ethics Committee of the Capital Region of Denmark (DNK), jr.no. H-16025210. All experiments have been performed in accordance with the Declaration of Helsinki^25^.

**Table 3:** Demographic overview of patient samples.

Brain	Origin	n	Sex		Age (mean ± SD)	RIN (mean ± SD)	PMI (mean ± SD)	DD (mean ± SD)
NC	6 BBH, 10 NBB	16	6M	10F	76.6 ± 10.9	5.2 ± 0.7	21.7 ± 20.9	
PSP	13 BBH, 3 HV	16	12M	4F	73.6 ± 8.2	5.5 ± 2.4	31.2 ± 17.1	7.9 ± 3.1
*p*			0.080		0.198	0.420	0.131	
**PBMC**								
NC	BMDB	18	14M	4F	70.1 ± 6.4			
PSP	BMDB	9	7M	2F	67.3 ± 7.9			7.3 ± 3.7
*p*			>0.999		0.336			

NC: Normal controls; PSP: Progressive supranuclear palsy; BBH: Bispebjerg Brain Bank; BMDB: Bispebjerg Movement Disorder Biobank; NBB: Netherlands Brain Bank; HV: Harvard Brain and Tissue Resource Center; M: Male; F: Female; RIN: RNA Integrity Number; PMI: Post-mortem interval; DD: Disease duration; PBMC: Peripheral blood mononuclear cells. Age and DD are shown in years, PMI is shown in hours. Age, RIN, PMI and DD are shown as mean ± standard deviation. DD is defined as time from first symptoms to death. *P* values for group differences are shown on the bottom.

### Protein analyses

Protein extraction and quantification of total levels were performed as earlier described^9^. In short, app. 50 mg brain tissue was disrupted and homogenized using a MagNA Lyzer Instrument (Roche Life Science) in a buffer containing protease inhibitors. Samples were spun to pellet cell debris. Sample concentrations were determined using the Bradford protein quantification assay^26^. The protein concentrations in the NC sample extracts (mean 3.61 ± SD 1.04 mg/ml) did not differ from the PSP sample extracts (mean 3.17 ± SD 0.56 mg/ml; Welch’s unpaired t-test, t = 1.421, *p* = 0.171). All samples were aliquoted to avoid thawing prior to analysis. Cytokine and neurotrophin protein levels were investigated on a Bio-Plex Pro Human Cytokine 17-plex Assay (BIO-RAD, USA; #M5000031YV) which included antibodies for the targets interleukin(IL)-1β, IL-2, ILs 4–8, IL-10, IL-12, IL-13, IL-17, granulocyte colony-stimulating factor (G-CSF), granulocyte macrophage colony-stimulating factor, interferon-gamma, monocyte chemoattractant protein 1, macrophage inflammatory protein(MIP)1β, and tumor necrosis factor(TNF)alpha(α). Further, the following singleplex targets were added to the assay: basic fibroblast growth factor (BIO-RAD; #171B5016M); MIP1α (BIO-RAD; #171B5022M); platelet-derived growth factor BB (BIO-RAD; #171B5024M); and vascular endothelial growth factor (VEGF; BIO-RAD; #171B5027M). The assays were analysed on a Bio-Plex 200 System (Luminex xMAP Technology, BIO-RAD) following the manufacturer’s instructions. Samples were added in a concentration of 1 mg protein/ml after 1:2 dilution in sample diluent.

Validation of IL-2 and G-CSF protein levels were investigated on MSD V-PLEX Human IL-2 Assays (Meso Scale Discovery (MSD), USA; #K151QQD; lower limit of detection (LLOD): 0.09 pg/ml) and MSD U-PLEX G-CSF Assays (MSD; #K151VGK; LLOD: 1.6 pg/ml), respectively, following the manufacturer’s instructions. Plates were analyzed on a SECTOR S 600 instrument (MSD).

### mRNA analyses

Extraction, quality assessment of total RNA, DNA contamination, and analysis of mRNA levels was performed as previously described^9^. All RNA experiments were performed in accordance with the MIQE guidelines^27^. Extraction of RNA was performed using the miRNeasy Mini Kit (Qiagen, NLD; #217004). In short, app. 30 mg brain tissue was homogenized by intensive pipetting in a lysis reagent. Samples were then phase separated in chloroform, and subjected to on-column DNase treatment. Samples were eluted in 30 µl nuclease-free water. RNA integrity was measured using the Agilent RNA 6000 Nano Kit (Agilent Technologies, USA; #5067-1511) on an Agilent 2100 Bioanalyzer (Agilent Technologies). All samples had a RIN >  3.95 as suggested earlier^28,29^. DNA contamination was investigated using primers for glyceraldehyde 3-phosphate dehydrogenase (*GAPDH*). cDNA synthesis was performed using qScript (Quanta BioSciences, USA; #95048) with 100 ng input RNA. cDNA concentrations were measured on a NanoDrop 2000C (Thermo Fisher Scientific), and diluted to 100 ng/µl in nuclease-free water. RT-qPCR was performed with SYBR Green Master Mix 2X (Thermo Fischer Scientific; #4309155) on a Stratagene Mx3005P qPCR system, or a QuantStudio 3 Real-Time PCR System (Thermo Fischer Scientific). All primers had an efficiency of 93–108% with an R^2^ > 0.98. Primer concentrations ranged from 300 to 500 nM with an annealing temperature of 56–62 °C. An acquisition step was added when necessary at 77–80 °C. The amplicons were 72 to 146 base pairs long. A melting curve was included in each reaction for product verification. Targets of interest were: glycogen synthase kinase 3β (*GSK3B*), forward primer: 5-ACAACAGTGGTGGCAACTCC-3, reverse primer: 5-TTCTTGATGGCGACCAGTTCT-3^29^; nuclear factor kappa-light-chain-enhancer of activated B cells inhibitor alpha (*NFKBIA*), forward primer: 5-AGCCTACAAGAAAGTTTGCCTAT-3, reverse primer: 5-TCTTCTTCCGGTAGTGGATCTTGGC-3; nuclear receptor subfamily 4 group A member 2 (*NR4A2*; also known as *Nurr1*), forward primer: 5-CCGGGATCTCTCCACAACTT-3, reverse primer: 5-GGAGACTGGCGTTTTCCTCT-3; and transcription factor p65 (*RELA*; also known as *NFKB-p65*), forward primer: 5-CTGCCGGGATGGCTTCTAT-3, reverse primer: 5-CCGCTTCTTCACACACTGGAT-3^30^. A calibrator sample (cDNA from Human Reference Total RNA; Clontech, USA; #636538), was included on each plate^31^. Sample cycle threshold (Ct) values were normalized to the reference genes^29^: ubiquitin-conjugating enzyme E2D2 (*UBE2D2*), forward primer: 5-TGCCTGAGATTGCTCGGATCT-3, reverse primer: 5-TCGCATACTTCTGAGTCCATTCC-3^32^; ribosomal protein 13a (*RPL13A*), forward primer: 5-AGCCTACAAGAAAGTTTGCCTAT-3, reverse primer: 5-TCTTCTTCCGGTAGTGGATCTTGGC-3; and topoisomerase 1 (*TOP1*), forward primer: 5-GGCGAGTGAATCTAAGGATAATGAA-3, reverse primer 5-TGGATATCTTAAAGGGTACAGCGAA-3^33^. Normalized values were calculated using the geometric mean^34^.

### Immunohistochemistry

Immunostainings on FFPE tissue samples were performed as previously described^9^. In short, brain samples from three PSP patients and three NCs containing both white and grey matter were excised and fixed in formalin for min. 48 h in 10% buffered formalin before embedding in paraffin on a Leica ASP300 S tissue processor (Leica, DEU). Samples were cut on a sliding microtome into 5 µm sections. All antibodies were tested alone using HRP and 3,3′-diaminobenzidine tetrahydochloride hydrate as previously described^9^. For immunofluorescent double labelling, slides were deparaffinized before antigen retrieval at pH 6 (NeuN) and/or 9 (IL-2, GFAP) followed by incubation with primary antibodies: Monoclonal rabbit anti-human IL-2 (1:250; Abcam; #ab92381), and monoclonal mouse anti-human NeuN (1:500; Merck Millipore; #MAB377) or monoclonal mouse anti-human GFAP (1:200; Dako; #M0761). Secondary antibodies were goat anti-mouse IgG Alexa Fluor 488 (1:200; Invitrogen; #A11001) and goat anti-rabbit IgG TRITC (1:1000; Abcam; #ab6718). Cover slides were mounted with medium containing DAPI. Stainings were investigated using a Nikon Eclipse 80i microscope. Specificity of the IL-2 antibody was tested on tonsils as a positive control (data not shown). Additionally, appropriate isotype controls were included to verify specificity.

### Flow cytometry

Blood was collected in 9 ml EDTA tubes (Greiner Bio-One; #455036). Briefly, blood was diluted 1:1 in Dulbecco’s PBS (DPBS; Sigma-Aldrich; #D8537) containing 2 mM EDTA (Sigma-Aldrich; #E9884) and 1:5 in a Ficoll-Paque PLUS gradient (GE Healthcare Life Science; #17144003). Cells were washed twice with PBS and frozen in RPMI-1640 (Sigma-Aldrich; #R5885) containing 40% fetal calf serum (FCS; Gibco; #26010-074) and 10% dimethyl sulfoxide (Sigma-Aldrich; #D4540) in cryotubes at −80 °C in a Mr. Frosty Freezing Container (Thermo Scientific), then stored at −130 °C until use.

On the day of analysis, PBMCs were thawed at 37 °C for 10 min before washing in 10% FCS in RPMI-1640 followed by centrifugation at 200 g for 10 min. 200,000 cells were added to a well on a round bottom plate. The plate was spun down at 2,600 RPM for 2 min before washing in 1X Foxp3 TF Wash Buffer (Invitrogen; A24261) in 1% BSA (Sigma-Aldrich; #05482) in PBS followed by another spin down. Cells were incubated for 30 min in the dark at 4 °C in 45 µl antibody staining solution. The staining solution contained antibodies against TCR-α/β (1:8; BD Biosciences; #555548), CD3ε (1:50; BioLegend; #300431), CD4 (1:50; BD Biosciences; #564976), CD8 (1:50; BD Biosciences; #565310), CD14 (1:25; BioLegend; #325618), CD16 (1:25; BioLegend; #302012), CD45R0 (1:50; BD Biosciences; #562327), CD45RA (1:50; BD Biosciences; #563963), CD56 (1:25; BioLegend; #318334), and CD57 (1:25; BD Biosciences; #561906) in Brilliant Stain Buffer (BD Biosciences; #563794). Cells were washed in 180 µl 1X Foxp3 TF Wash Buffer and centrifuged at 600 × g for 5 min before 150 µl 1X Fixation and Permeabilization Solution (Buffer A: Invitrogen; #A24217; Buffer B: Invitrogen; #A24218) was added for 15 min at room temperature. After centrifugation at 600 × g for 5 min, cells were resuspended in 200 µl 1% BSA in PBS for analysis.

Samples were quantified on a BD LSR II flow cytometer (BD Biosciences). A minimum of 20,000 events were counted per sample. Data were analyzed using FlowLogic v. 7.2.1 (Inivai Technologies). The gating strategies are illustrated in Supplementary Figs. S1 and S2 which are based on compensation and FMO analyses.

### Statistics

ILs 4, 5, 8, 10, 12, 17, MIP1α, TNFα and VEGF proteins were omitted from the analyses as detection sensitivity was less than 50%. One sample did not present detectable protein levels in any measurements on the Luminex assays and was excluded from further analyses. For each target, samples that presented measurements below the minimum detection limit were omitted from the analysis. Outliers were identified using the ROUT method with Q = 1% in GraphPad Prism v. 7.02 (GraphPad Software Inc., USA). Demographic data were analyzed using an unpaired t-test (age), a Mann-Whitney test (RNA Integrity Number (RIN) and post-mortem interval (PMI)), or Fischer’s exact test (sex). Spearman’s Rank-Order Correlation test was applied to correlation analyses of clinical characteristics and IL-2 protein levels. Student’s t-test or Welch’s t-test was used for flow cytometry analyses. The remaining statistical analyses were performed in R v. 3.4.1^35^. Normality was assessed using the Shapiro-Wilk Normality test. Scedasticity and collinearity were assessed using *ncvTest* and *vif*, respectively, from the *car* package^36^. If necessary, data were log10-transformed. Data were analyzed using multiple linear regression with models that included relevant variables that have previously been shown to possibly impact results^9^. These variables included group (NC vs. PSP), age, sex (female vs. male), and PMI as well as RIN for the mRNA targets. Familywise error rate was adjusted using Bonferroni correction for the multiplex data (adj. *p* = 4.17*10^−3^, n = 12). Graphs were made in GraphPad Prism v. 7.02.

## Supplementary information

Supplementary Figures

## Data Availability

The datasets generated and analysed during the current study are available from the corresponding author on reasonable request.
