# Supplementary material for: Increased prefrontal cortex interleukin-2 protein levels and shift in the peripheral T cell population in progressive supranuclear palsy patients
Source: Sci Rep. 2019 May 23;9:7781. doi: 10.1038/s41598-019-44234-y (PMC6533275; doi:10.1038/s41598-019-44234-y)
Supplement: Supplementary file 1 — Supplementary Figures [file 41598_2019_44234_MOESM1_ESM.docx]

# Title

Increased prefrontal cortex interleukin-2 protein levels and shift in the peripheral T cell population in progressive supranuclear palsy patients

# Authors and affiliations

Rasmus Rydbirk*^1^, Betina Elfving^2^, Jonas Folke^1^, Bente Pakkenberg^1^_,_^3^_,_ Kristian Winge^3^_,_^4^, Tomasz Brudek^1^, Susana Aznar^1^

^1^Research Laboratory for Stereology and Neuroscience, Bispebjerg-Frederiksberg Hospital, University Hospital of Copenhagen, Nielsine Nielsens Vej 6B, stair 11B, 2^nd^ floor, DK-2400, Copenhagen NV, Denmark

^2^Translational Neuropsychiatry Unit, Department of Clinical Medicine, Aarhus University, Skovagervej 2, DK-8240, Risskov, Denmark

^3^Institute of Clinical Medicine, Faculty of Health, University of Copenhagen, Blegdamsvej 3B, DK-2200, Copenhagen, Denmark

^4^Department of Neurology, Bispebjerg-Frederiksberg Hospital, University Hospital of Copenhagen, Bispebjerg Bakke 23, DK-2400, Copenhagen, Denmark

*Corresponding author: Rasmus Rydbirk, Nielsine Nielsens Vej 6B, stair 11B, 2^nd^ Floor, DK-2400 Copenhagen NW, Denmark, [rasmus.rydbirk@regionh.dk](mailto:rasmus.rydbirk@regionh.dk)

# Supplementary Figure S1


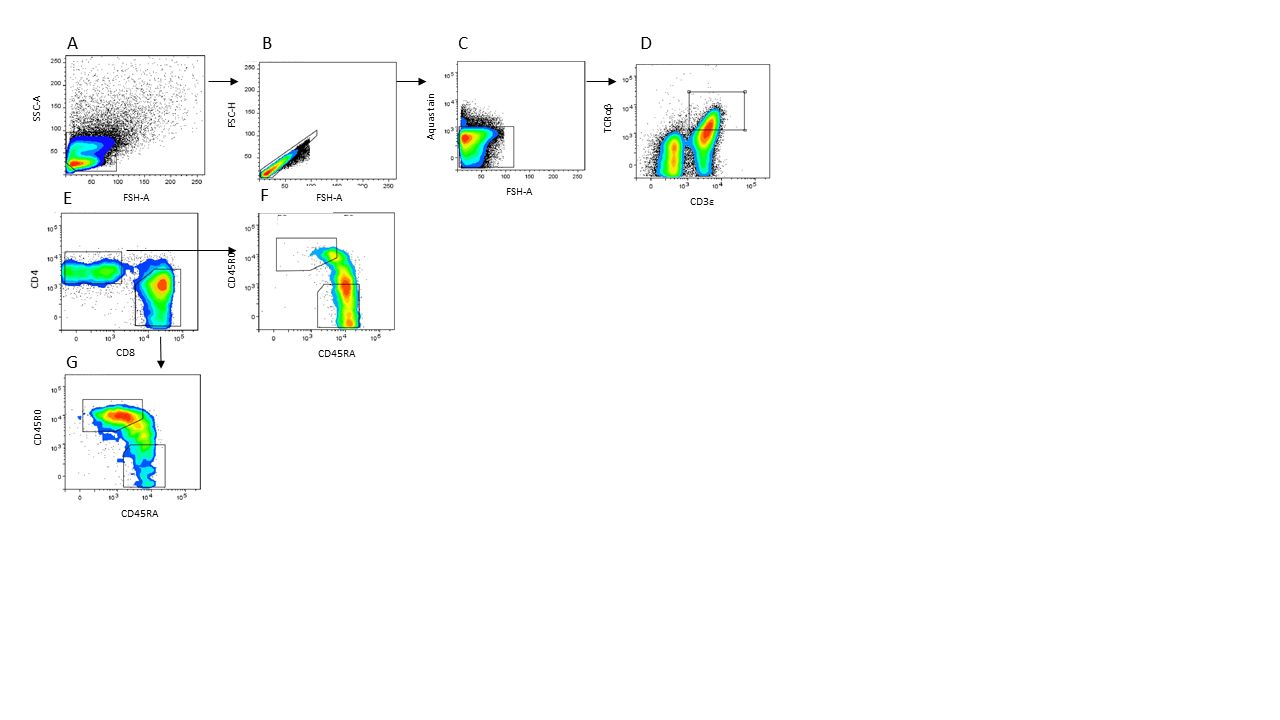


**Supplementary Figure S1 – T-cells gating strategy.** The leukocyte population was chosen (**A**) before gating for single cells (**B**), live cells (**C**) and TCRαβ^+^CD3ε^+^ cells (**D**). Two populations were chosen (**E**), either CD4^-^CD8^+^ cells (**F**), or CD4^+^CD8^-^ cells (**G**). Both populations were analysed for their expression of CD45R0 and CD45RA (**F** and **G**).

# Supplementary Figure S2 – NK cell gating strategy


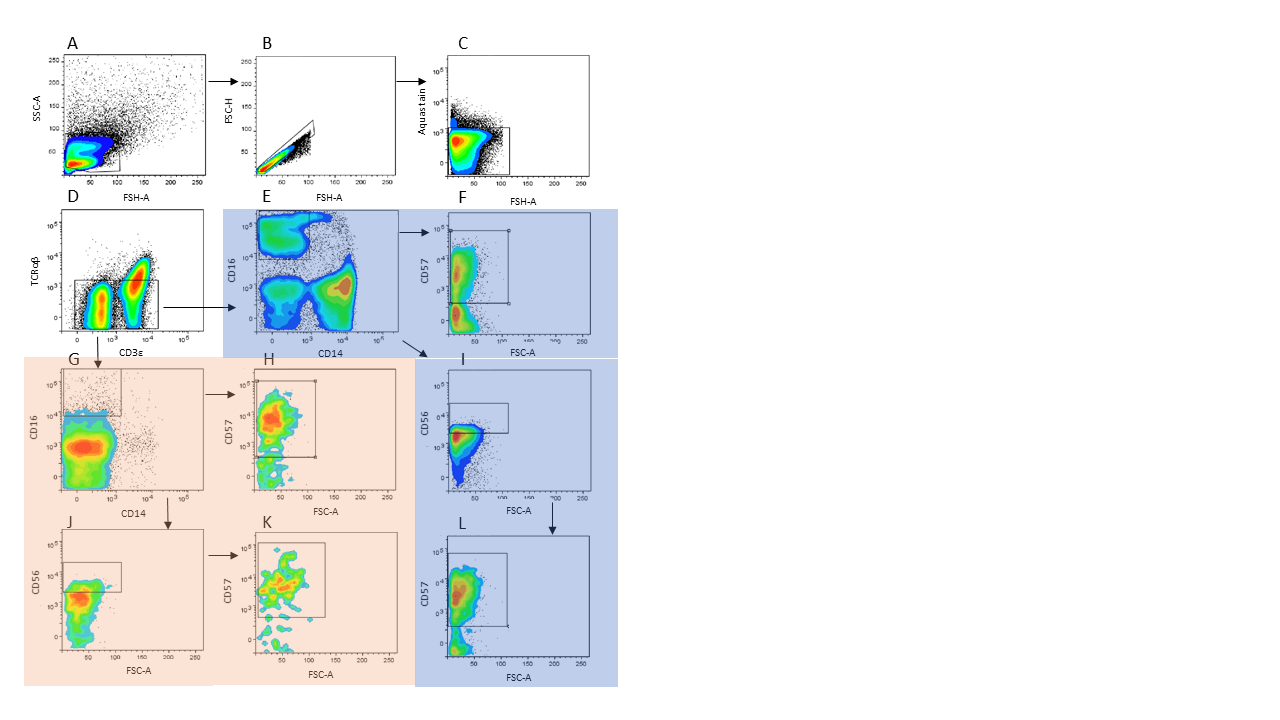


**Supplementary Figure S2 – NK-cells gating strategy.** The leukocyte population was chosen (**A**) before gating for single cells (**B**) and live cells (**C**). Two populations were chosen (**D**), either TCRαβ^-^CD3ε^+^ cells (**E**, **F**, **I**, **L**; blue background), or TCRαβ^-^CD3ε^-^ cells (**G**, **H**, **J**, **K**; red background). For both populations, CD14^-^CD16^+^ cells (**E**, **G**) were analysed for expression of CD57 (**F**, **H**), or CD56 (**I**, **J**) and then CD57 (**K**, **L**).

# Supplementary Figure S3 – Correlations to clinical characteristics


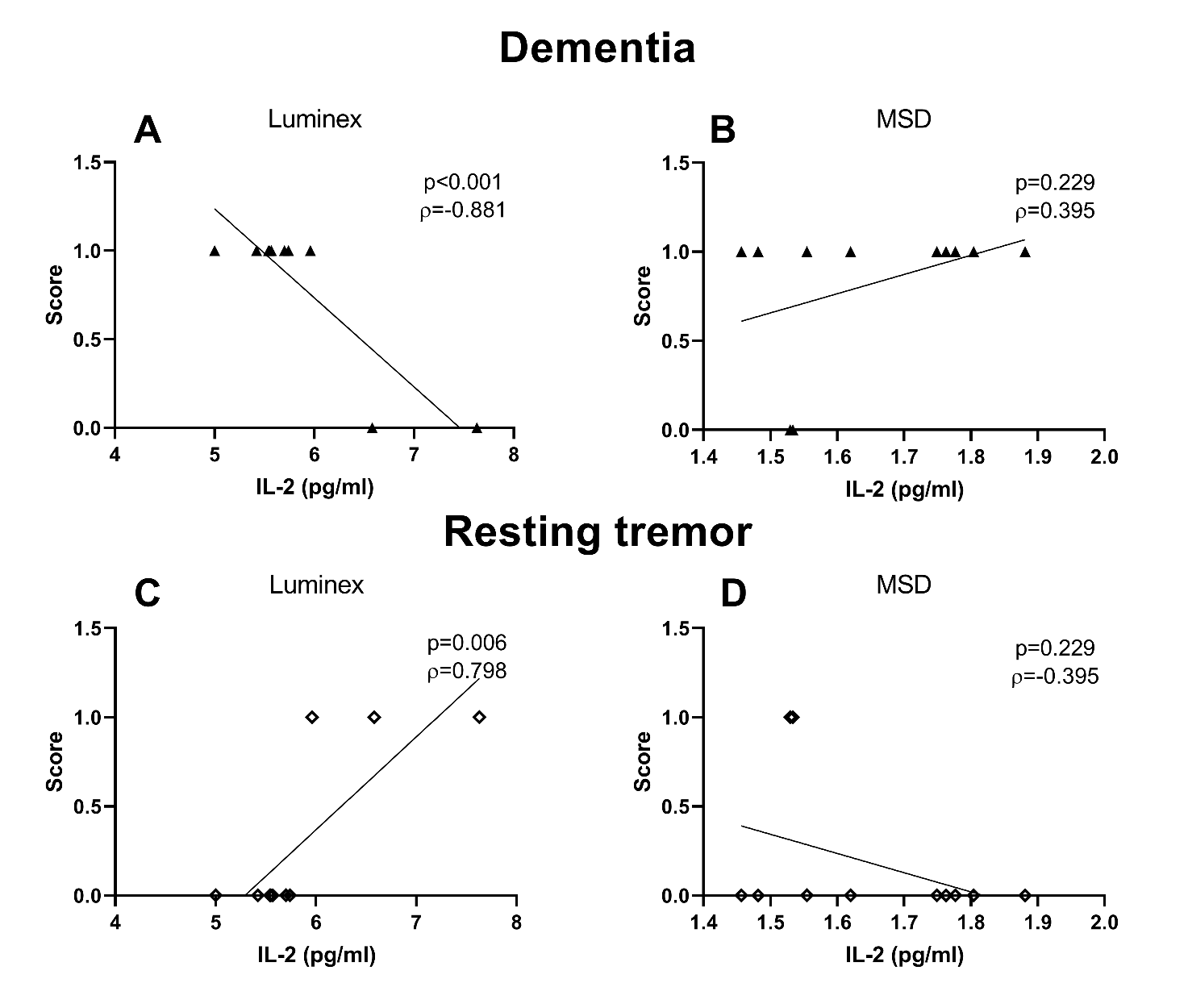


**Supplementary Figure S3 – Correlations to clinical characteristics. A** and **B** show correlation of interleukin-2 (IL-2) protein levels to the presence of dementia within the first year after diagnosis; **C** and **D** show correlation of IL-2 protein levels to the presence of resting tremor within the first year after diagnosis; **A** and **C** show correlations to Luminex measurements; **B** and **D** show correlations to MSD measurements. Data were analysed using Pearson correlation.
